# Long-Term Outcomes and Prognostic Factors in Advanced Gallbladder Cancer: Focus on the Advanced T Stage

**DOI:** 10.1371/journal.pone.0166361

**Published:** 2016-11-15

**Authors:** Chen Chen, Zhimin Geng, Haoxin Shen, Huwei Song, Yaling Zhao, Guanjun Zhang, Wenzhi Li, Li Ma, Lin Wang

**Affiliations:** 1 Department of Hepatobiliary Surgery, First Affiliated Hospital of Xi'an Jiaotong University, Xi’an, Shaanxi, China; 2 Department of Epidemiology and Biostatistics, School of Public Health, Xi’an Jiaotong University, Health Science Center, Xi’an, Shaanxi, China; 3 Department of Pathology, First Affiliated Hospital of Xi'an Jiaotong University, Xi’an, Shaanxi, China; Yonsei University College of Medicine, REPUBLIC OF KOREA

## Abstract

**Background:**

Radical resection is an effective therapeutic method to increase the survival rate of patients with gallbladder cancer (GBC). In addition to the surgical approach, the relationships between various clinicopathologic factors and the outcome of patients with GBC remain controversial.

**Methods:**

Clinical and laboratory examination characteristics, pathological and surgical data, and post-operative survival time of 338 patients with advanced GBC who received treatment at the First Affiliated Hospital of Xi'an Jiaotong University, China from January 2008 to December 2012 were analyzed retrospectively. Factors influencing the prognosis of GBC after surgery were analyzed by univariate and multivariate analysis.

**Results:**

The overall survival rates for curative resection patients were significantly greater than those for non-curative resection patients (1-,3-,5-year survival rate and mean-survival time: 59.0%, 47.3%, 44.3% and 22.0 months vs. 12.7%, 8.3%, 7.7% and 3.0 months) (P < 0.001). For the curative resection patients, positive margin, lymph node metastasis, poorly pathological differentiation and the presence of ascites were all independent risk factors for poor prognosis. For patients with T3 stage, neither segmentectomy of IVb and V nor common bile duct resection improved the prognosis (P = 0.867 and P = 0.948). For patients with T4 stage, aggressive curative resection improved the prognosis (P = 0.007).

**Conclusions:**

An advanced T stage does not preclude curative resection. Positive margin, lymph node metastasis, poorly pathological differentiation and the presence of ascites are all independent risk factors for poor prognosis in the curative intent resection patients. The range of liver resection and whether common bile duct resection is performed do not influence the prognosis as long as R0 resection is achieved.

## Introduction

Gallbladder cancer (GBC) is the most common malignant tumor of the biliary system, presenting features such as high degree of malignancy, difficult early diagnosis, poor therapeutic effects and prognosis, and with a dismal survival rate of 0–12% in most reports [1]. The global rates for GBC exhibit striking variability, reaching epidemic levels for some regions and ethnicities. The basis for this high degree of variability likely resides in differences in environmental exposures and intrinsic genetic predisposition to carcinogenesis [2,3]. The morbidity rate from GBC and cholangiocarcinoma in Chinese cancer registration areas was 4.31/100,000, and the population-standardized incidence rate was 1.93/100,000 [4], a rate which was equal to global levels.

Radical resection has been shown to be an effective therapeutic method to increase the 5-year survival rate in patients with GBC [5]. Unfortunately, most of patients with GBC have lost an opportunity for radical resection when visiting, less than 10 percentage of patients have tumors that can be resected at the time of surgery. The 5-year survival rate for 131 GBC patients subjected to surgical treatment was 13 percentage in Taner's original report, and patients that underwent a radical cholecystectomy had a significantly longer median survival (24 months) than patients that had a simple cholecystectomy (6 months) or non-curative treatment (4 months) [6].

For T1a GBC, the optimal treatment method is simple cholecystectomy, which can be carried out as either a laparotomy or a laparoscopic surgery. For T1b GBC, extended cholecystectomy is appropriate. An extended cholecystectomy is generally recommended for patients with GBC at stage T2 or above. In extended cholecystectomy, a wedge resection of the gallbladder bed or a segmentectomy of IVb/V can be performed and the optimal extent of lymph node dissection should include the cystic duct lymph node, the common bile duct (CBD) lymph node, the lymph nodes around the hepatoduodenal ligament (the hepatic artery and portal vein lymph nodes), and the posterior superior pancreaticoduodenal lymph node. Depending on patient’s status and disease severity, surgeons may perform palliative surgeries [7]. For T4 disease, extended cholecystectomy is not sufficient to achieve negative margin, extended radical resection, such as hepatopancreatoduodenectomy (HPD), especially major hepatectomy (resection of 2 or 3 hepatic sections) with pancreatoduodenectomy, have recently received increasing attention in the treatment of advanced GBC, and have shown curative potential with negative margins, even in patients with advanced GBC [8,9].

However, factors influencing the prognosis of patients with GBC include liver involvement [10], lymphatic metastases [11] and jaundice [12] are still in dispute. Some surgical techniques like the range of liver resection and CBD resection are also inconclusive, and the efficacy of aggressive surgical resection for T4 GBC has not been accepted generally [13,14].

In the present study, the clinical and laboratory examination characteristics, pathological and surgical data as well as post-operative survival time of 338 patients with advanced GBC were analyzed retrospectively. The purpose of the present study was to investigate the factors influencing prognosis and to evaluate the different surgical procedures for advanced GBC.

## Methods

### Patients and data collection

After screening against exclusion criteria including unclear diagnosis, no surgical treatment and no follow up evaluation, 338 patients with advanced GBC who received treatment at the First Affiliated Hospital of Xi'an Jiaotong University, China from January 2008 to December 2012 were retrospectively reviewed. Data including sex, age, and clinical manifestation were collected. Jaundice was defined by the serum bilirubin level exceeding 34.2 μmol/L (2 mg/dL). Ascites was defined as more than 100 mL of fluid built up within the peritoneal cavity during the surgery. Clinical end-points and measurements included (1) imaging examination data such as abdominal ultrasound, computed tomography (CT) and magnetic resonance (MR) scan, and (2) serological tumor markers, including carbohydrate antigen 125(CA-125), carbohydrate antigen 19-9(CA19-9) and carcinoembryonic antigen(CEA), and (3) details of the surgical method and other surgical data. The pathological evaluation and GBC diagnosis were re-analyzed according to the defining criteria published in 2010 by World Health Organization(WHO) [15]. Patients were assessed for tumor/node/metastasis (TNM) staging according to the American Joint Committee on Cancer (AJCC) (7th edition) [16].

### Indications for surgery

Different surgical procedures were used according to the results of exploratory surgery and intraoperative pathological examination. In patients with advanced GBC without involvement of the liver or minimal infiltration into the liver, wedge resection of the gallbladder bed/segment IVb/V resection and regional / extend lymph node (LN) dissection was planned. When the massive invasion of the liver was diagnosed, major hepatectomy, such as right hemihepatectomy or right trisectionectomy was indicated. If the tumors involved extrahepatic bile duct or bulky regional lymph node metastasis around the bile duct was found, CBD resection was added. Peritoneal seeding, bulky lymph node involvement, or para-aortic lymph node involvement was regarded to be contraindications for surgery. HPD was considered in patients with the following conditions: (1) lower bile duct involvement, (2) pancreatic infiltration, (3) duodenal infiltration, and (4) bulky retropancreatic lymph node metastasis. Gastric resection was performed in case of macroscopic infiltration.

Palliative surgical interventions were performed when en bloc tumor removal cannot be achieved because of distant metastases, peritoneal seeding, positive para-aortal lymph nodes, or wide tumor invasion, or body conditions cannot afford aggressive surgery or patients refused. For the palliative surgery, biliary tract drainage was performed once jaundice or biliary tract invasion occurred.

### Ethics Statement

The study was approved by the Ethics Committee of the First Affiliated Hospital of Xi'an Jiaotong University,China. All patients gave written informed consent to participate. The ethics committee approved this consent procedure. The data did not contain any information that could identify the patients.

### Follow-up

Clinical follow-up was scheduled at the first, third, sixth and twelfth month after discharge, and subsequently once a year during which, the deadline for following up was October 2014. Overall survival (OS) was defined as the time interval from the date of surgery to the date of death. Follow-up data were obtained from outpatient clinic visits, phone calls, and questionnaires submitted by mail.

### Statistical analysis

Results were analyzed using the Statistical Package for the Social Sciences for Windows version 13.0 (SPSS Inc, Chicago, IL)software program. Measured data were described as the mean ± standard deviation (SD), and comparisons between groups were done using the Student’s t-test. Enumeration data were expressed by percentage, and comparisons between groups were analyzed with the chi-square test. Kaplan-Meier survival curves were plotted and Log-rank statistics were calculated to assess which of the variables affected the survival time. Survival analysis was analyzed with the Kaplan–Meier method, differences were measured with the Log-rank test, and prognostic multivariate analysis was analyzed by COX regression. A level of p < 0.05 was considered statistically significant.

## Results

### Clinical and pathological data

There were 102 males, 236 females, and the proportion of male and female patients was 1:2.3. The age range was from 29 to 86 years, and the mean age was 62.0 ± 10.3 years. Right epigastralgia was the main presenting clinical symptom, accounting for 75.7% (256 patients). Jaundice was present in 25.1% (85 patients) and epigastric lump was seen in 5.6% (25 patients). 205 patients (60.7%) were complicated with cholecystolithiasis, 27 patients (8.0%) were complicated with diabetes mellitus, and 61 patients (18.0%) were accompanied with hypertension. CA19-9 showed the highest positive rate (60.3%) in preoperative tumor marker examination. Tumor were mainly located in the bottom and body regions, accounting for 34.9% and 27.2% of cases respectively. Adenocarcinoma was seen in 85.8% in pathological type, and a poor state of differentiation was the main pathological differentiation type. In 58.0% of patients, GBC was accompanied with liver involvement, wherein 74.9% of cases co-presented with lymph node metastasis, and28.1% of the cases were accompanied with distant metastases.

### Surgical Procedure

Curative intent resection was performed in 134 patients, including (1) cholecystectomy, wedge resection and regional LN dissection (n = 52), (2) cholecystectomy, wedge resection and extended LN dissection (n = 18), (3) cholecystectomy, segmentectomy IVb/V and regional LN dissection (n = 22), (4) cholecystectomy, segmentectomy IVb/V and extended LN dissection (n = 19), (5) HPD (n = 9), (6) cholecystectomy, wedge resection, regional lymph node dissection and subtotal gastrectomy (n = 2), and (7) major hepatectomy, cholecystectomy and regional LN dissection (n = 12). Resection margin status was determined as R0 or R1 (R0 = no residual disease, R1 = microscopic residual disease).

Non-curative resection was performed in 204 patients (60 subjected to cholecystectomy, 95 cholecystectomy and biliary tract external drainage, 7 cholecystectomy and internal biliary drainage, 21 biliary tract external drainage alone, 14 exploratory laparotomy and 7 gastrointestinal anastomosis). Detailed clinical and pathological data of curative and non-curative resection is described in Table 1.

**Table 1:** Clinical and pathological data of patients with GBC.

		Cases (percentage)		P-value
		Curative resection	Non-curative resection	
Sex	Male	38	64	0.555
	Female	96	140	
Age, y	<50	46	76	0.200
	51–70	66	82	
	>70	22	46	
CA19-9	Positive	30	87	**<0.001**
	Negative	45	32	
CA-125	Positive	27	61	**0.026**
	Negative	41	46	
CEA	Positive	26	54	**0.033**
	Negative	63	70	
Tumor location	Bottom	36	32	0.092
	Body	27	26	
	Neck	10	28	
	Bottom and body	11	17	
	Neck and body	3	4	
	Cystic duct	1	0	
Pathological type	Adenocarcinoma	120	170	0.109
	Non-Adenocarcinoma	14	34	
UGC	No	99	186	**<0.001**
	Yes	35	18	
Pathological differentiation	Well	13	13	**0.029**
	Moderately	67	79	
	Poorly	54	112	
Liver involvement	Yes	44	152	**<0.001**
	None	90	52	
Jaundice	Yes	18	67	**<0.001**
	None	116	137	
T stage	T3	123	87	**<0.001**
	T4	11	117	
N stage	N0	66	19	**<0.001**
	N1	41	90	
	N2	27	95	
M stage	M0	134	109	**<0.001**
	M1	0	95	
TNM stage	IIIA	64	16	**<0.001**
	IIIB	35	2	
	IVA	8	45	
	IVB	27	141	
Blood type	A	36	50	0.926
	B	46	76	
	AB	14	20	
	O	31	44	
Ascites	Yes	15	65	**<0.001**
	None	119	139	

UGC = unsuspected gallbladder carcinoma; CA-125 = carbohydrate antigen 125; CA19-9 = carbohydrate antigen 19–9; CEA = carcinoembryonic antigen.

Boldface: Significant values (P < 0.05)

During the operation, 80 patients (23.7%) were confirmed to have an accompanying ascites. There were 53 patients with unsuspected gallbladder carcinoma (UGC) (31 IIIA, 7 IIIB, 3 IVA and 12 IVB). Among these patients, 18 patients were diagnosed during or after cholecystectomy in our hospital (14 IIIA, 2 IIIB, 1 IVA and 1 IVB), and they had a better tumor staging comparing with other GBC patients (49 IIIA, 30 IIIB, 50 IVA and 156 IVB) (P < 0.001).

### Overall survival rate

**Fig 1:**
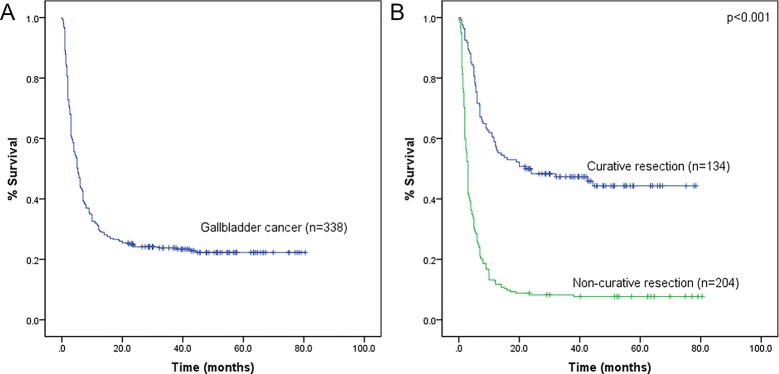
Overall survival curve of GBC patients. (A): General overall survival curve; (B): Overall survival curve of R0 and R1/2 resected patients: P< 0.001.

The median survival time was 5.2 months, and the 1-, 3- and 5-year overall survival rate were 31.1%, 23.8%, and 22.3% respectively (Fig 1A).

The 1-, 3-, and 5-year overall survival rates for curative intent resection patients were 59.0%, 47.3%, and 44.3%, respectively, and the median survival time was 22.0 months. These survival rates were significantly higher than those in patients not subjected to non-curative resection (12.7%, 8.3%, 7.7%, and 3.0 months, respectively) (P < 0.001) (Fig 1B).

### Curative intent resection

A total of 134 patients underwent curative intent resection. The detailed surgical procedures performed according to T stage are described in Table 2. There were 18 patients R1 (positive biliary margin in 7 patients and positive liver margin in 11 patients), including T3N0M0 in 4 patients, T3N1M0 in 7 patients and T3N2M0 in 7 patients. Factors including demographic characteristics (sex, age, blood type), underlying diseases (e.g., hypertension, and diabetes mellitus) and differential clinical pathological characteristics (preoperative jaundice, gallstone, tumor location, presence of ascites, liver involvement, T stage, lymph node metastasis, TNM stage, margin, intraoperative blood loss, type of pathology and pathologic differentiation degree, UGC) of patients subjected to curative intent resection were compared by the Log-rank test.

**Table 2:** Surgical procedures according to T stage.

		T stage	
		3	4
Surgical procedures	Cholecystectomy, wedge resection + standard LN dissection	52	0
	Cholecystectomy, wedge resection+ extended LN dissection	18	0
	Cholecystectomy, segmentectomy IVb/V+ standard LN dissection	22	0
	Cholecystectomy, segmentectomy IVb/V+ extended LN dissection	19	0
	Hepatopancreatoduodenectomy	0	9
	Cholecystectomy, wedge resection+ regional LN dissection and subtotal gastrectomy	0	2
	Major hepatectomy, cholecystectomy + regional LN dissection	12	0

The results showed that liver involvement, margin, the presence of ascites, TNM stage, T stage, N stage, and pathological differentiation were significant risk factors that influenced the prognosis (Table 3).

**Table 3:** Univariate analysis of prognosis for curative intent resection GBC patients.

Factor		Cases	Median survival time (m)	P-value	Survival rate (%)		
					1 year	3 years	5 years
Gallstone	None	53	24.0	0.682	60.4	48.2	48.2
	Yes	81	20.0		58.0	46.6	41.8
Hypertension	None	112	24.0	0.593	59.8	48.6	45.0
	Yes	22	16.0		54.5	40.9	40.9
Diabetes mellitus	None	120	20.0	0.982	59.2	48.0	44.4
	Yes	14	24.0		57.1	42.9	42.9
Age, y	<50	46	Not reach	0.065	69.6	57.6	57.6
	50–70	66	11.7		50.0	39.3	33.6
	>70	22	20.0		63.6	50.0	50.0
Sex	Male	38	32.0	0.578	63.2	48.0	48.0
	Female	96	19.0		57.3	46.7	42.9
Blood type	A	36	13.0	0.335	52.8	41.7	33.3
	B	46	12.3		54.3	41.3	38.4
	AB	14	Not reach		64.3	64.3	64.3
	O	31	Not reach		64.5	53.4	53.4
Jaundice	None	116	24.0	0.362	60.3	48.7	45.4
	Yes	18	9.0		50.0	38.9	38.9
Position	Bottom	36	Not reach	0.299	66.7	55.1	55.1
	Body	27	Not reach		63.0	55.3	55.3
	Neck	10	Not reach		80.0	60.0	60.0
	Bottom and body	11	7.5		36.4	27.3	27.3
	Neck and body	3	Not reach		66.7	66.7	66.7
	Cystic duct	1	7.0		0	0	0
Ascites	None	119	42.6	**0.001**	63.9	50.8	47.3
	Yes	15	6.0		20.0	20.0	20.0
UGC	No	99	16.0	0.606	56.6	45.2	45.2
	Yes	35	42.6		65.7	53.3	40.0
Margin	R0	116	Not reach	**<0.001**	64.7	53.8	50.4
	R1	18	5.0		22.2	5.6	5.6
Intraoperative blood loss	<1000	121	32.0	0.194	60.3	49.2	46.0
	≥1000	13	11.7		46.2	30.8	30.8
Liver involvement	None	90	Not reach	**0.001**	67.8	57.6	53.4
	Yes	44	9.3		40.9	26.3	26.3
TNM stage	IIIA	64	Not reach	**<0.001**	90.6	81.1	75.0
	IIIB	35	7.0		40.0	28.3	28.3
	IVA	8	11.0		37.5	0	0
	IVB	27	5.7		14.8	7.4	7.4
T stage	3	123	42.6	**0.005**	61.0	51.1	47.8
	4	11	11.0		36.4	0	0
N stage	0	66	Not reach	**<0.001**	89.4	78.7	72.7
	1	41	8.0		39.0	23.7	23.7
	2	27	5.7		14.8	7.4	7.4
Pathological differentiation	Well	13	Not reach	**0.009**	92.3	84.6	75.2
	Moderately	67	42.0		62.7	50.3	47.3
	Poorly	54	9.0		46.3	34.6	34.6
Pathological type	Adenocarcinoma	120	24.0	0.258	60.8	48.8	45.5
	Non-adenocarcinoma	14	10.0		42.9	35.7	35.7

UGC = unsuspected gallbladder carcinoma.

Boldface: Significant values (P< 0.05)

A multivariate analysis was performed to determine prognostic relationships were independent predictive factors. TNM stage was excluded in multivariate analysis on account relating with T stage and N stage. The results showed that the positive margin, lymph node metastasis, poorly pathological differentiation and the presence of ascites were all independent risk factors for poor prognosis. These findings indicate that neither liver involvement nor advanced T stage in patients with GBC influenced postoperative survival after curative intent resection in accordance with adequate criteria (Table 4).

**Table 4:** Results of COX multivariate regression analysis.

Variable	Regression coefficient	Standard error	p-value	Relative risk	95% Confidence interval
Ascites	0.706	0.331	**0.033**	2.025	1.059–3.873
Margin	1.413	0.325	**<0.001**	4.108	2.172–7.770
Liver involvement	0.431	0.308	0.162	1.539	0.842–2.812
T stage	0.518	0.386	0.180	1.679	0.788–3.578
N stage	0.983	0.178	**<0.001**	2.673	1.887–3.787
Pathological differentiation	0.476	0.222	**0.032**	1.609	1.041–2.487

UGC = unsuspected gallbladder carcinoma.

Boldface: Significant values (P< 0.05)

#### Liver and CBD resection in R0 resection T3 patients

**Fig 2:**
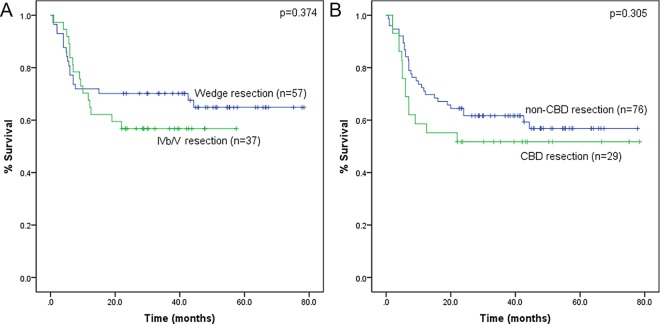
Overall survival curve of T3 GBC patients with different surgical techniques. (A): Wedge resection vs. segmentectomy IVb/V: P = 0.374. (B): CBD resection VS non-CBD resection: P = 0.305.

After exclusion of 18 patients with R1 margin, there were 105 patients with T3 accepted R0 resection, 11 patients underwent major hepatectomy because of major liver invasion. Among the remaining 94 patients, 57 patients underwent a wedge resection and 37 underwent segmentectomy of IVb/V. Median survival time was not achieved in both groups, the 1-, 3- and 5-year overall survival rates were 71.9%, 70.2% and 64.9% for wedge resection and 67.6%, 56.8% and 56.8% for IVb/V resection (P = 0.374) (Fig 2A).

Twenty-nine patients out of 105 T3 patients with R0 resection underwent CBD resection. The median survival time was not achieved in both the CBD resection group and the non-CBD resection group, the 1-, 3- and 5-year overall survival rates were 58.6%, 51.7% and 51.7% for CBD resection group and 71.1%, 61.7% and 56.8% for non-CBD resection group (P = 0.305) (Fig 2B).

#### Aggressive curative resection in T4 patients

R0 resection was carried out in 9.1% (11 of 128) of patients with T4, including HPD in 9 patients and subtotal gastrectomy in 2 patients. There were 2 T4N0M0, 6 T4N1M0, 3 T4N2M0, and 1 T4N2M0, 1 patient was alive at the end of the follow up with the survival time of 21.9 months, the survival for the other patients were 3.0, 3.3, 5.0, 7.5, 11.0, 11.7, 12.2, 12.3, 32.0 months, respectively, and 1 T4N2M0 patient died of pancreatic fistula at 1.6 months after HPD. The median survival time was 11.0 months for the curative resection group and 2.3 months for non-curative resection group, the 1-, 3- and 5-year overall survival rate were 36.4%, 0% and 0% for the curative resection group, and 6.8%, 3.4% and 1.7% for the non-curative resection group (P = 0.007).

## Discussion

GBC is an aggressive cancer and the majority of the patients with GBC remain asymptomatic or have vague complaints in the early stage of the disease [17], thus the early preoperative diagnosis of GBC is still difficult and most patients present with advanced disease(T3-T4) at diagnosis. Curative resection may be the only available method to cure patients presenting with GBC [18]. It has been widely considered a critical factor influencing the prognosis of GBC in many reports [10,19–22]. Curative resection should be performed according to TNM staging. Distant metastasis has been accepted as a contraindication of radical resection. Surgical treatment has been well established for T1 and T2 GBC. However, whether advanced T stage and lymph node metastasis precludes radical resection is still in dispute. According to the most recent TNM staging definition, T3 disease is worthy of radical resection, but T4 disease is usually considered to be unlikely to benefit from surgical resection and should be treated with palliative therapies [21,23]. However, there is no consensus regarding unresectable factors in local extension of biliary tract cancers currently [24], and several recent reports have shown improved prognoses in patients with these locally advanced cancers following surgical resection combined with arterial resection, reconstruction, or extended trisectionectomy of the liver and HPD [13,24]. T4 GBC resection is becoming acceptable in patients where R0 surgery is achievable [20]. Nishio et al. [25] concluded that GBC involving the extrahepatic bile duct was worthy of resection. Agarwal et al. [26] also reported that duodenal infiltration was not indicative of no resectability in terms of HPD. Birnbaum et al.[20] also stated that N status predicted outcome, while T status was not a prognostic indicator in locally advanced GBC. Similarly, in the present study, curative resection significantly improved the prognosis of GBC patients, indicating that advanced T stage does not preclude curative resection, and aggressive surgical intervention is suitable for the locally advanced GBC without distant metastasis, even if the lesion involves neighboring organs. It was widely reported that the prognosis of UGC patients was closely related to the tumor staging, but not to the type of surgical approach, and the first operation did not affect the outcome no matter it was LC or OC [23,27–31]. Moreover, the time of diagnosis also has no adverse effect on the outcome of patients with UGC, regardless of whether the tumor is detected during or after cholecystectomy [32]. In this study, our finding also confirms that UGC does not influence the prognosis once curative resection is achieved.

Previous studies have found that preoperative jaundice indicates a poor prognosis, and resection cannot improve the prognosis of GBC co-presenting with jaundice [12,21]. Thus preoperative jaundice should be considered a relative contraindication to radical resection for GBC [33]. However, recent reports have demonstrated declared that although preoperative jaundice indicates poor prognosis and high postoperative morbidity, it does not preclude radical resection, especially in highly selected patients (N0) [34,35], and aggressive surgery might improve long-term survival in advanced GBC patients with obstructive jaundice [36]. In this study, patients with obstructive jaundice who underwent curative resection had overall survival that were similar to those in patients without obstructive jaundice, and jaundice at diagnosis showed no prognostic impact once R0 was achieved. Ascites worsens the prognosis. In this study, 15 curative resection patients present with ascites, which was associated with hypoproteinemia. The serum albumin level was affected by not only liver function and nutrition but also the systemic inflammatory response. Patients with advanced GBC sometimes develop cholangitis or treatment-resistant cholecystitis, which may result in hypoalbuminemia [37]. Albumin is one of the independent prognostic factors for overall survival in other cancers such as pancreatic cancer and gastric cancer [38,39]. Therefore, preoperative ultrasound examination and correcting hypoalbuminemia are very important. However, abdominal paracentesis and ascites cytopathology examination or laparoscopy should be performed to exclude malignant ascites when necessary.

Lymph node status is always one of the strongest predictors of survival [40,41]. We also confirm lymph node metastasis is one of the independent risk factors for curative resection in GBC patients. However, there is no consensus on the lymphadenectomy in the management of GBC. Some authors believe that the positive nodes of N2 station did not preclude a curative resection [20,42,43], whereas in most cases, N2 metastasis was interpreted as remote metastasis and GBC patients with N2 metastases have not been thought to benefit from aggressive surgery generally [21,44–46]. Therefore, radical lymph node dissection should not be routinely performed [47–49]. Further research, especially RCT research, should be carried out to figure out whether distant lymph node metastasis is a contraindication for radical surgery.

R1 resection was associated with 0% survival at 3 years in the Nagoya series and Birnbaum’s report[20,25]. In the present study, the 5-year survival rate of R1/2 resection was only 5.6%. Therefore, R1 resection is also the most important factor associated with poor prognosis in multivariate analysis. Most of the 18 R1 patients received treatment in 2008–2009 and at that time we did not realize the importance of frozen pathological sample examination of the margin, which should be routinely performed in each curative intent resection. For the T4 disease, CBD resection is usually performed during aggressive surgery such as HPD. In this study, we analyzed the impact of CBD resection in T3 patients and found that patients with and without CBD resection had similar OS. CBD resection did not yield a higher lymph node count and was not associated with an improved survival [10]. This result was also confirmed by Gani in a study involving 449 GBC patients [50]. Therefore, with Increasing risk of surgical trauma and postoperative complications, routine CBD resection is not recommended and should only be performed when CBD invasion and positive margin of the cystic duct occurred. We also found that there were no significant differences in survival between liver wedge resection and segmentectomy IVb/V in T3 patients. According to a previous study by Araida [51], cumulative survival rate does not differ between these two kinds of liver resection. So either of these hepatic surgical procedures is feasible as long as R0 margin is provided.

This study has several limitations. First, we cannot get the disease-free survival time of these patients, limiting the statistical power of this study. Second, the effects of postoperative chemotherapy and radiotherapy on prognosis were not considered. Actually, adjuvant therapy for patients with advanced GBC following surgical resection are meaningful options and should be recommend. The range of chemotherapy includes gemcitabine, fluoropyrimidines or gemcitabine-based combination chemotherapy[33]. In a latest multi-institutional analysis, adjuvant therapies were reported independently associated with improved long-term outcomes in GBC patients with advanced T stage, LN metastasis and R1 margin[52], and a similar conclusion was also declared by Ma et al. in a meta-analysis[53]. However, very few patients would like to receive these adjuvant therapies in our district so that we could not analyze the relationship between adjuvant therapies and prognosis in this research. Therefore, supplementary studies involving a larger number of patients and focusing on the extent of surgery based on lymph node metastasis and adjuvant therapies are needed in the future.

In conclusion, this study retrospectively analyzed the clinical data of 338 patients with advanced GBC who received surgical treatment. We confirm that an advanced T stage does not preclude curative resection. Positive margin, lymph node metastasis, poorly pathological differentiation and the presence of ascites are all independent risk factors for poor prognosis in patients subjected to curative intent resection. The range of liver resection and whether CBD resection is performed do not influence the prognosis as long as R0 is achieved. This study attempted to provide a reference for evaluating the survival time of patients with advanced GBC, and improving the surgical therapy for GBC.

## Supporting Information

S1 Table Dataset of 338 patients with GBC underwent surgery. (XLSX)
